# Targeting bone homeostasis regulation: potential of traditional Chinese medicine flavonoids in the treatment of osteoporosis

**DOI:** 10.3389/fphar.2024.1361864

**Published:** 2024-04-02

**Authors:** Jiazhe Du, Yincang Wang, Chengliang Wu, Xinyu Zhang, Xiaofeng Zhang, Xilin Xu

**Affiliations:** ^1^ Graduate School, Heilongjiang University of Traditional Chinese Medicine, Harbin, China; ^2^ Institute of Orthopedics and Traumatology, The First Affiliated Hospital of Zhejiang Chinese Medical University, Hangzhou, China; ^3^ Teaching and Research Section of Orthopedics and Traumatology, Heilongjiang University of Chinese Medicine, Harbin, China; ^4^ Department of Orthopedics, The Third Affiliated Hospital of Heilongjiang University of Traditional Chinese Medicine, Harbin, China

**Keywords:** flavonoids, osteoporosis, traditional Chinese medicine, bone homeostasis, flavonols

## Abstract

Osteoporosis is a systemic metabolic disease characterized by disrupted bone formation/resorption and homeostasis. Flavonoids extracted from traditional Chinese medicinal plants regulate bone homeostasis by intervening in differentiating bone marrow mesenchymal stem cells, balancing the bone immune system, inhibiting oxidative stress response, and reversing iron overload. The target molecules and signaling pathways, such as Wnt/β-catenin and OPG/RANKL/RANK, directly affect osteoblast/osteoclast activity, exhibiting significant potential in the treatment of OP. Therefore, this study presents a systematic review of the recent literature to provide comprehensive information on the traditional Chinese medicine flavonoids involved in the regulation of bone homeostasis. Also, the molecular mechanisms and pharmacological uses of these metabolites are summarized, and their clinical translation and development potential are discussed.

## 1 Introduction

Osteoporosis (OP) is a systemic bone metabolism disease characterized by decreased bone mineral density, destruction of bone microstructure, and an increased risk of fractures. The incidence of the disease is closely related to age, and primary OP is most common in postmenopausal and senile conditions. As the global population ages, the incidence of this disease is increasing (Zhu et al., 2023).

**FIGURE 1:**
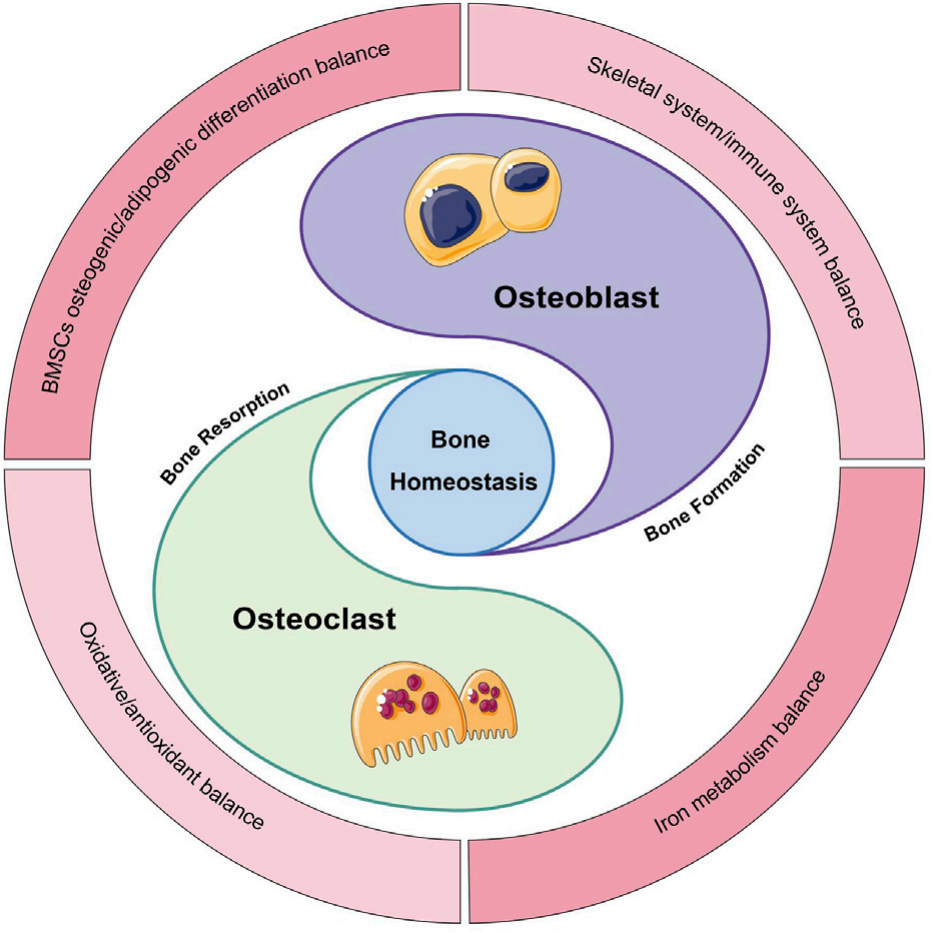
Manifestations of bone homeostasis. Bone homeostasis is a state of balance between bone formation and bone resorption, closely related to OBs/OCs, and has several manifestations.

Regarding the mechanism of occurrence, the bone remodeling imbalance theory suggests that the bone resorption rate is greater than the rate of bone formation, which disrupts the body’s normal homeostasis, ultimately leading to the development of OP. This process is effectuated by multicellular bone, the basic functional unit. Also, the damaged bone is resorbed, forming new bone to fill the holes. The unit is mainly composed of osteoblast (OB) and osteoclast (OC) (Loundagin and Cooper, 2022). OB produces new bone, and OC dissolves old bone to mediate bone formation and resorption, respectively, maintaining a dynamic balance for the stability of the skeletal system. Therefore, optimal bone homeostasis is regarded as the balance between OB and OC activities. Literature review identified several manifestations of homeostasis, such as bone marrow mesenchymal stem cells (BMSCs) osteogenic/adipogenic differentiation balance, skeletal system/immune system balance, oxidative/antioxidative balance, and iron metabolism balance (Figure 1).

Over the years, numerous therapeutic drugs have been developed for OP to improve bone metabolism. Bisphosphonates, calcitonin, oestrogens, their receptor modulators, monoclonal antibodies, and tissue protease inhibitors are used to inhibit bone resorption, while parathyroid hormone and active vitamin D are employed to promote bone formation (Morin et al., 2023). These drugs, whether used alone or in sequential combination, have demonstrated efficacy in regulating bone homeostasis. However, their clinical use is limited due to high costs, adverse effects, and potential carcinogenicity. For instance, bisphosphonates, the most common first-line therapy for OP, work by binding to bone to prevent resorption. Alendronate and zoledronic acid are notable examples. Alendronate often causes esophageal discomfort and gastric dyspepsia, whereas zoledronic acid typically leads to flu-like symptoms and fever in its acute phase (Lu et al., 2020). In addition, hormone therapy is frequently employed during early menopause to counteract bone loss caused by estrogen deficiency. Large doses of exogenous estrogen therapy can increase the risks of breast and endometrial cancer, cardiovascular events, and thromboembolism (Rozenberg et al., 2020). So long-term use of this is discouraged. In recent years, there has been a gradual increase in basic research on the use of traditional Chinese medicine (TCM) for the treatment of OP. TCM exhibits fewer adverse effects compared to conventional first-line drugs, making it suitable for prolonged use. Studies have shown that TCM, either alone or combined with first-line drugs, offers superior clinical efficacy.

Several studies have screened many active metabolites from Chinese herbal plants; flavonoids comprise a significant category. In terms of avoiding adverse drug reactions, citrus bioflavonoids seem to be safe, without causing side effects even during pregnancy; naringin-derived drugs are used in traditional medicine because of their non-addictive and non-toxic properties (Bampidis et al., 2022); isoflavones not only help reduce the risk of breast and endometrial cancer but also alleviate hot flushes associated with menopause (Gómez-Zorita et al., 2020). Yong et al. (2021) conducted a double-blind experiment on 58 healthy postmenopausal women, administering a daily dose of Epimedium prenylflavonoid extract (740 mg) or a placebo for 6 weeks. Based on verifying the association between the drug and bone synthesis metabolic markers, they further demonstrated that taking the drug was not associated with adverse symptoms, with no observed changes in liver, hematological, and renal parameters. Compared to first-line medications, natural flavonoid metabolites are mostly similar to estrogens, possessing anti-inflammatory, antibacterial, anticancer, antioxidant, osteogenic, osteoclast, and estrogen-like effects (Cushnie and Lamb, 2005). They can regulate disease and body health from a variety of perspectives, not just limited to regulating bone metabolism. This is one of the advantages of these metabolites. In addition, there is a growing body of research demonstrating the superiority of flavonoids alone or in combination with first-line drugs (Oršolić et al., 2014; Liu et al., 2015; Oršolić et al., 2022). This type of research is still scarce, mostly based on animal experiments, and involving clinical evidence is even more rare. Some researchers conducted a randomised double-blind trial among healthy late postmenopausal women (Zhang et al., 2007). They found a significant reduction in bone loss in the intervention group (a daily dose of 60 mg of icariin, 15 mg of daidzein, and 3 mg of genistein). In another clinical study of 360 osteoporosis patients, Epimedium total flavone capsule showed higher efficacy in back pain, leg pain scores, and BMD enhancement rates compared to Gusongbao capsule (Lu et al., 2013). Though there are some flavonoid supplements currently sold as TCM products, wellcharacterised extracts studied through rigorously designed clinical trials are still rare. In addition, a growing body of evidence is suggesting that TCM flavonoids can modulate OB and OC activities to regulate bone homeostasis. Herein, we conducted a comprehensive systematic review on the potential of TCM flavonoids in treating OP by restoring bone homeostasis.

## 2 Characterization of flavonoids

**FIGURE 2:**
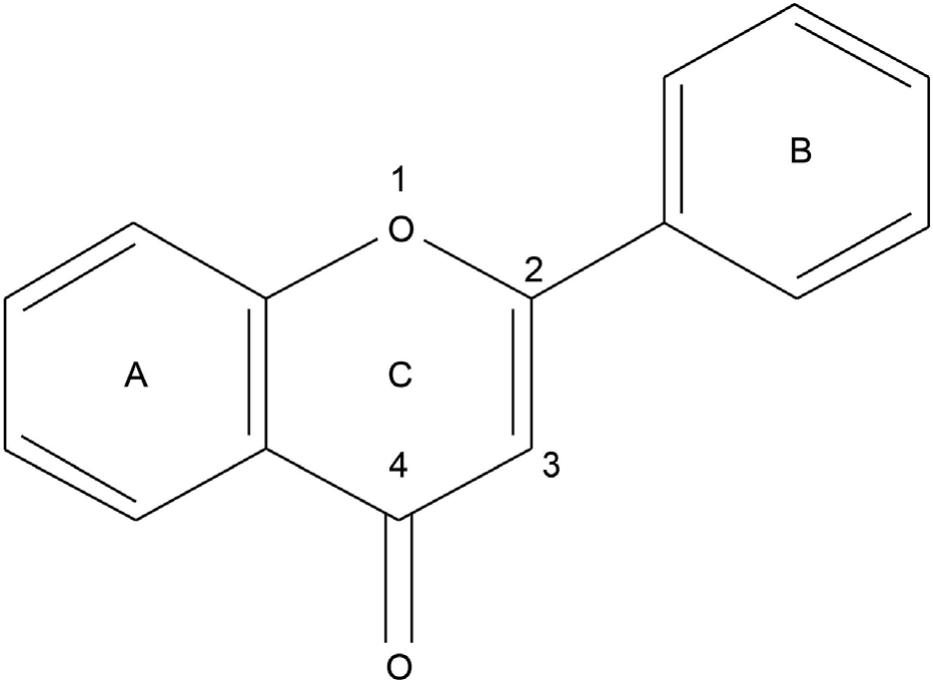
Parent nucleus structure of flavonoids.

As a subclass of polyphenols, flavonoids possess unique chemical structures and biological functions. They, also known as bioflavonoids or plant flavonoids, are predominantly present in food-borne plants, such as vegetables, fruits, legumes, and tea, either in bound or free form (Yonekura-Sakakibara et al., 2019). Structurally, flavonoids refer to a series of compounds in which two phenolic hydroxyl benzene rings (A- and B-ring) are linked to each other by the three central carbon atoms. The basic parent nucleus is 2-phenylchromanone (Figure 2). Approximately, 10,000 types of flavonoids can be categorized based on their structures into flavones, flavonols, flavanones, flavanonols, isoflavones, and others (Yonekura-Sakakibara et al., 2019). Among these, flavones do not have oxygen-containing groups at the “3” position, but if there is an “-OH” or other oxygen-containing groups at the “3” position, they are known as flavonols. Flavanones and flavanonols are based on flavones and flavonols, respectively. The double bond at the C2-C3 position of the C ring is hydrogenated and saturated and hence, termed as dihydroflavones and dihydroflavonols. In the case of isoflavones, the B ring position is connected to the “3” position rather than the “2” position. Regarding stability, it is now generally accepted that flavonoids enhance stability through the conjugation of the C2-C3 double bonds and acyl groups, as well as electronic delocalization. The α and β unsaturated pyranones at the centre of the molecule are the key to their various biological activities. In general, the strength of antioxidant and other biological activities of flavonoids is determined by the number of phenolic hydroxyl groups. The higher the number of substituents, the stronger the activity of scavenging oxygen radicals, especially the neighbouring hydroxyl substitution can greatly increase the activity. Regarding reactivity, the reactivity of flavonoids towards specific targets is dependent on the bioactive hydroxyl group in the structure (He et al., 2021). The Fukui index confirms this by identifying molecule regions susceptible to nucleophilic, electrophilic, and radical attacks (Ayers and Parr, 2000). Regarding drug activity, the broad-spectrum pharmacological activities of flavonoids have been a hotspot for researchers. Demonstrated pharmacological activities include antitumor, antioxidant, anti-inflammatory, antibacterial, and antiviral effects, among others (Guan and Liu, 2016; Shen N. et al., 2022). They can also alleviate cardiovascular diseases such as hyperlipidaemia and myocardial ischaemia. Their mild phytoestrogenic properties render them promising for treating menopausal syndrome in women (Miksicek, 1993).

Up to now, more than 9,000 flavonoids have been isolated from plants. As key constituents of many TCMs, some flavonoids can be extracted directly from TCM plants. However, these metabolites exhibit low solubility, poor stability, and limited bioavailability. Additionally, plant extracts typically comprise complex mixtures. To enhance flavonoids' bioavailability, recent years have seen the adoption of new extraction techniques, including ultrasound and supercritical fluid extraction. Moreover, enhancing activity through the synergistic effects of metabolites extracted from the same plant has garnered attention. Tomofumi et al. (Shimizu et al., 2018) demonstrated that baicalein, wogonin, and oroxylin A, extracted from scutellaria root, synergistically potentiate LPS-induced PGE2 and NO. Further, these metabolites uniquely inhibit various steps in the NF-κB signaling pathway. This synergistic effect also extends to the interaction between flavonoids and minerals. Bone minerals like calcium and phosphorus form hydroxyapatite, a crucial bone component also reflected in blood levels. Trace minerals, including magnesium, silicon, and zinc, directly influence bone metabolism. At this stage, some studies have proved that flavonoids can form metal complexes with metal ions or combine with metal nanomaterials to enhance antioxidant and anticancer activities (Matsia et al., 2022; Sysak et al., 2023; Zangade et al., 2023). This process also somewhat enhances the bioavailability of flavonoids.

**FIGURE 3:**
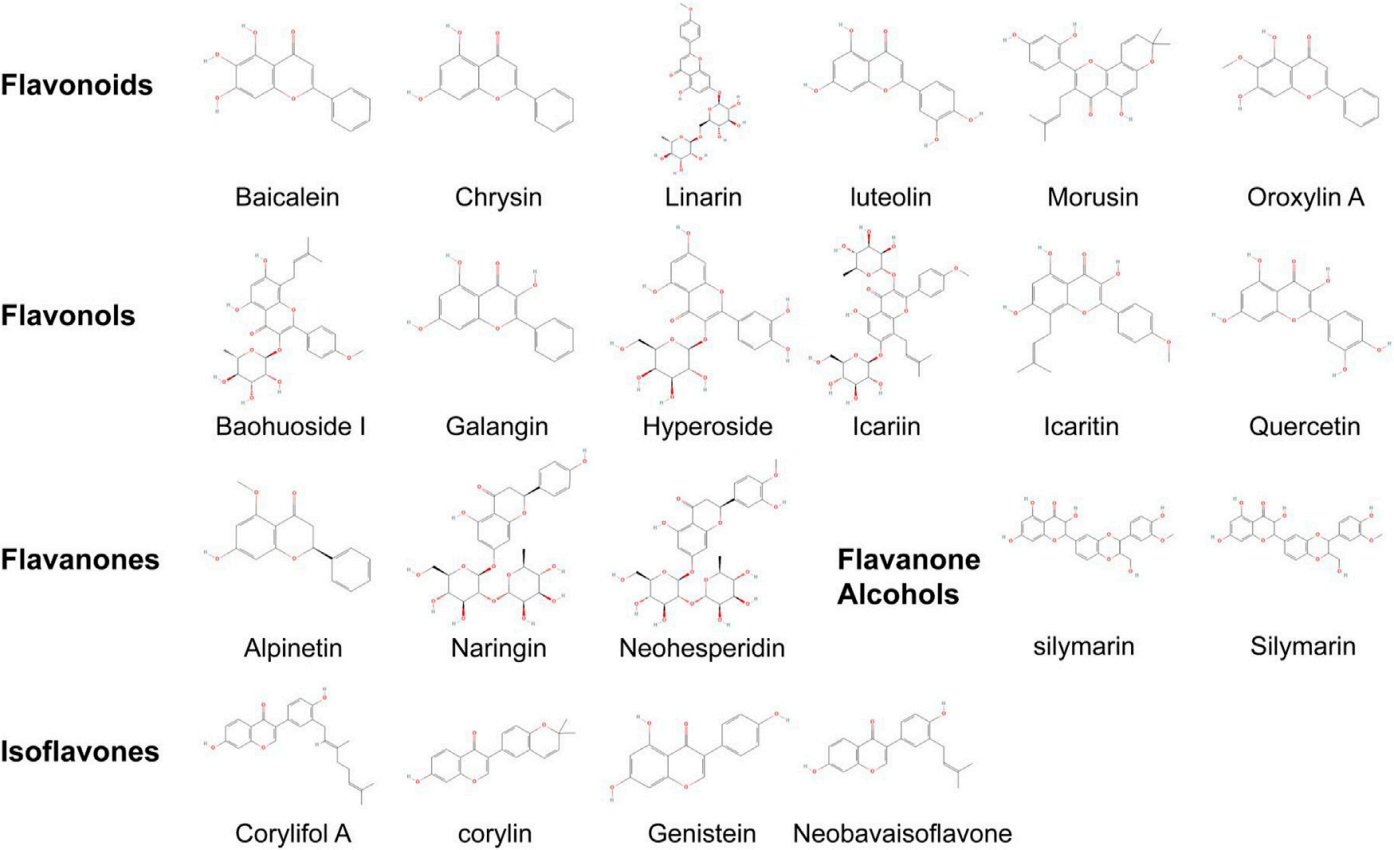
Structural formulas and classification of common TCM flavonoids. The compounds listed in this paper can be classified according to the presence or absence of oxygen-containing group substitution at the “3” position, whether the double bond is hydrogenated or not, and the position of the B-ring connection. They can be mainly divided into flavones, flavonols, flavanones, flavanonols and isoflavones.

In the treatment of OP, the anti-OP activity of flavonoids makes them safe and ideal natural therapeutic agents. They offer cost-effectiveness with minimal adverse effects (Guan and Liu, 2016). Previously, there have been a large number of herbal single drugs and compound preparations containing flavonoids that have been shown to have significant therapeutic effects on OP(Zhang et al., 2016). Over the years, a large number of active metabolites have been screened from them, especially total flavonoids of *Rhizoma drynariae* (Shen Z. et al., 2022), total flavonoids of *Epimedium*, and soy isoflavone (Ming et al., 2013). These flavonoids' active metabolites include naringin, icariin, and daidzein. Figure 3 illustrates common TCM flavonoids, highlighting their structural formulas.

## 3 TCM flavonoids modulate bone homeostasis

**FIGURE 4:**
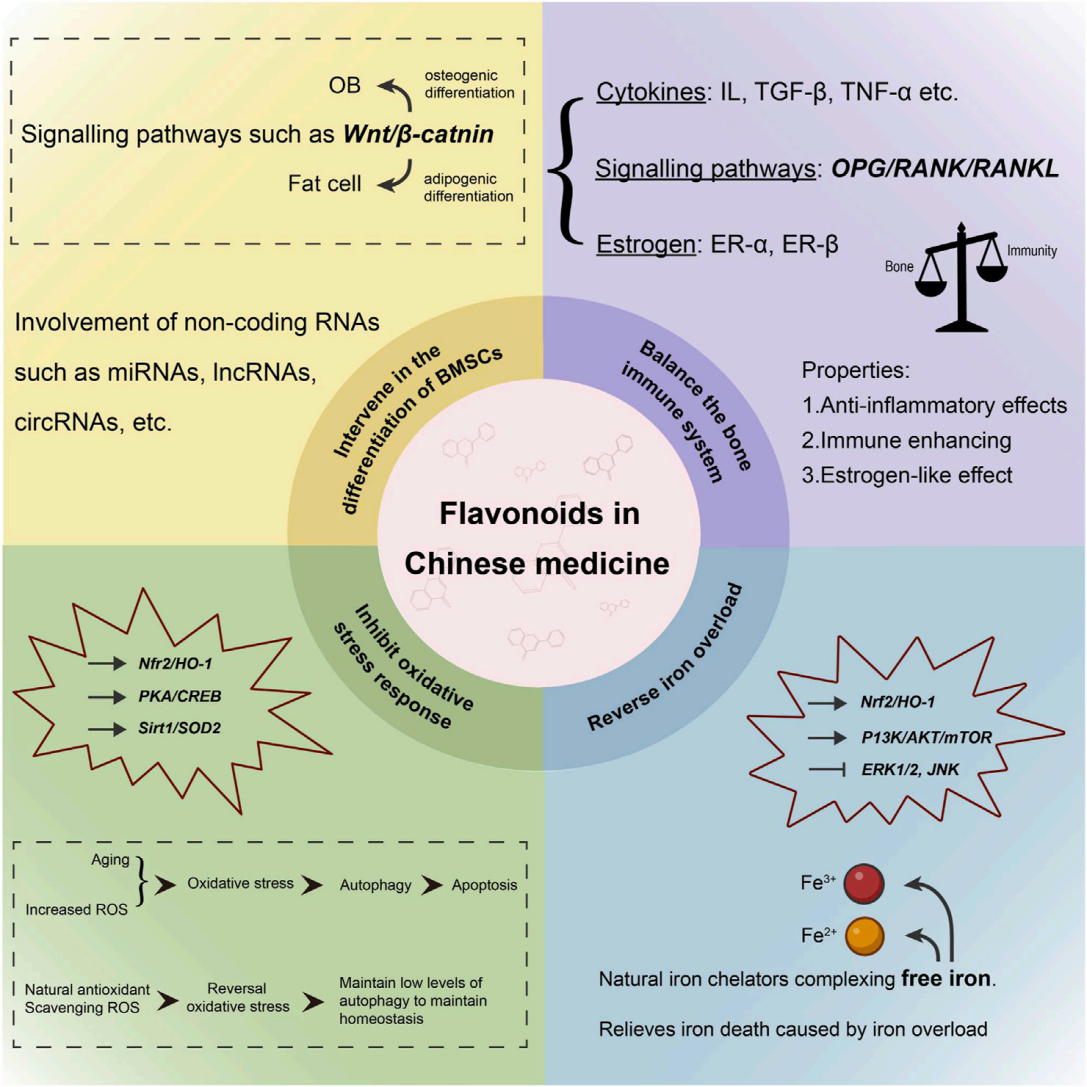
Pathways of TCM flavonoids regulating bone homeostasis.

Bone homeostasis can manifest in various forms, and the balance disruption can cause OP. The addition of TCM flavonoids can specifically regulate this process by intervening in the differentiation of BMSCs, balancing the bone immune system, inhibiting oxidative stress response, and reversing iron overload. The specific pathways are illustrated in Figure 4.

### 3.1 Intervention in the differentiation of BMSCs

Bone homeostasis is regulated by BMSCs’ osteogenic/adipogenic differentiation balance. In addition, BMSCs can differentiate into OBs and other cells, promote bone marrow tissue repair, and influence the activities of OBs and OCs. This provides a stable internal environment for bone growth and development. Typically, the bone regeneration process is closely related to the balance in osteogenic and adipogenic progenitor cell activities. An imbalance in osteogenic/adipogenic differentiation may arise if the number, proliferative capacity, and differentiation potential of bone progenitor cells decrease due to aging or specific physiological conditions. As a result, the bone homeostatic system regulated by BMSCs will be broken. In terms of mechanism, the classical Wnt signaling pathway, consisting of Wnt/β-catenin, has been found to be closely linked to bone formation. This pathway regulates OB differentiation and also acts as an inhibitor of adipocyte differentiation. The β-catenin protein in this pathway effectuates differentiation into OBs by inhibiting the adipogenic differentiation of BMSCs (Mei et al., 2022). Recent studies have focused on non-coding RNAs, including miRNAs, lncRNAs, and circRNAs, of which some regulate OP through the Wnt/β-catenin pathway (Liu et al., 2021; Wang Y. et al., 2022; An F. et al., 2023). They serve as diagnostic markers for disease and help expand the research on related target genes to further clarify the pathogenesis.

Nowadays, the study of TCM has advanced to the protein and gene level of pathways, elaborating the regulation of bone homeostasis via BMSCs’ osteogenic/adipogenic differentiation balance with respect to signaling pathway. Among the pathways closely related to the directional differentiation of BMSCs, Wnt/β-catenin has become a key target for OP intervention using botanical drug extracts. TCM flavonoids, such as neohesperidin, icariin, and morusin, regulate the differentiation of BMSCs into OB through this pathway (Chang et al., 2021; Chen et al., 2021; Gao et al., 2021). Icariin, a pentenyl flavonol glycoside isolated from *Epimedium brevicornu Maxim*. [Berberidaceae; epimedii folium], is one of the active metabolites in the treatment of OP and has been studied in depth more than others. One study suggested that the inhibition of BMSC differentiation may be associated with the overexpression of *sclerostin* gene or the transfection treatment of knockdown constructs, and treatment with icariin can reverse this process through the Wnt/β-catenin pathway (Gao et al., 2021). In addition to the investigation of the classical pathway, Wnt proteins activate non-classical signaling pathways through different receptors. However, these pathways are more complex and involve a larger number of species compared to the Wnt/β-catenin pathway. For example, Yuan et al. (2018) proposed that the NF-κB/β-catenin signaling pathway regulates BMSCs’ osteogenic/adipogenic differentiation balance. Quercetin, a flavonols, reversed these effects by inhibiting this signaling, significantly improving ovariectomized rats’ bone structure and function. Similar studies have been conducted based on other signaling pathways (for example, PKA/mTOR/ULK1, JAK2/STAT3, and PKA/CREB) that have elucidated the mechanism of action of some TCM flavonoids in the intervention of BMSCs’ differentiation (Wang W. et al., 2022; Zeng et al., 2023a; Zeng et al., 2023b). Among these, alpinetin and galangin attenuated dexamethasone-induced bone loss in mice with OP. Meanwhile, the osteogenic differentiation-inducing effects of these two drugs are related to enhanced autophagic signaling. Alpinetin enhanced the PKA/mTOR/ULK 1 autophagy signaling pathway, while galangin enhanced the PKA/CREB-mediated autophagy flux. In the study on the former, researchers first constructed a mouse model of OP and used parathyroid hormone as a positive control to verify that alpinetin significantly attenuated dexamethasone-induced OP symptoms. The study showed that a daily dose of 40 mg/kg body weight caused no toxic side effects, had negligible effects on OC activity, and initially alleviated OP symptoms. Next, to quantify the expression of osteogenic markers after alpinetin stimulation, osteocalcin (OCN), alkaline phosphatase (ALP), and Runx2 were measured. Combined with the previous experience they finally came to a conclusion that the preventive effect of alpinetin against OP was mediated by augmenting PKA/autophagy signaling because it was suppressed by co-treatment with a PKA inhibitor or autophagy inhibitor. In addition to pathway-specific studies, it was proposed that icariin promotes BMSC differentiation by upregulating miR-335-5p, suggesting that specific miRNAs can also be used as targets for the drug treatment of OP (Teng et al., 2022). This finding has expanded the scope for subsequent research. In addition, there have also been studies comparing TCM flavonoids with first-line drugs in terms of efficacy. For example, compared with the antiresorptive drug, alendronate, and an anabolic drug, PTH1-34, icarrin possessed the positive effects on the co-culture by increasing ALP activity, estradiol production and decreasing acid phosphatase (TRAP) activity (Liu et al., 2015). This goes some way to affirming the need to target flavonoids for R&D at this stage. The relevant studies on the intervention of TCM flavonoids in BMSCs differentiation are summarized in Table 1.

**TABLE 1:** Studies related to the intervention in the differentiation of BMSCs by TCM flavonoids.

Compound	Source	Model	Mechanism	Result	References
Icariin	*Epimedium brevicornu Maxim.*	12-week-old female SD rats	Activate the Wnt/β-catenin pathway; Regulate sclerostin; Enhance ALP activity	Promote *in situ* proliferation and osteogenic differentiation of BMSCs	Gao et al. (2021)
		Mouse MC3T3-E1 cells and RAW 264.7 cells	Enhance OPG and RANKL gene and protein expression and decrease NF-kB and RANK expression; Increase ALP and TGF-β1 levels	Promote osteogenic differentiation and inhibit adipogenic differentiation of BMSCs; Promote OB differentiation and inhibit OC differentiation	Zhang et al. (2017)
		7-week-old female ovariectomized SD rats	Upregulate miR-335-5p to inhibit PTEN	Promote osteogenic differentiation of BMSCs	Teng et al. (2022)
		Human BMSCs	Enhance GLI-1 expression	Promote proliferation and osteogenic differentiation of BMSCs	Xia et al. (2023)
		6-week-old female ovariectomized SD rats	Induce the IGF-1 pathway; Interaction with ER-α to phosphorylate downstream AKT	Promote osteogenic differentiation of BMSCs	Zhou et al. (2021)
Neohesperidin	*Citrus × aurantium f. aurantium*	Human BMSCs	Activate the Wnt/β-catenin pathway	Promote osteogenic differentiation of BMSCs	Chang et al. (2021)
Morusin	*Morus alba L.*	16-week-old female ovariectomized Wistar rats	Activate the Wnt/β-catenin pathway	Promote proliferation and osteogenic differentiation of BMSCs	Chen et al. (2021)
Quercetin	*Styphnolobium japonicum (L.) Schott*	Adult female ovariectomized SD rats	Antagonize the TNF-α-induced NF-κB/β-catenin signaling pathway	Promote osteogenic differentiation of BMSCs	Yuan et al. (2018)
Alpinetin	*Alpinia hainanensis K.Schum.*	Human BMSCs; mouse BMSCs	Enhance the PKA/mTOR/ULK1 autophagy signaling	Promote osteogenic differentiation of BMSCs	Zeng et al. (2023a)
Galangin	*Alpinia officinarum Hance*	3-month-old hormone-treated female C57BL/6 mice; human BMSCs	Enhance PKA/CREB autophagy signaling	Promote osteogenic differentiation of BMSCs	Zeng et al. (2023b)
Naringin	*Citrus × aurantium f. aurantium*	Female Lewis rats	Inhibit activation of the JAK2/STAT3 pathway	Promote osteogenic differentiation of BMSCs	Wang et al. (2022a)
Total flavonoids of *Rhizoma drynariae*	*Drynaria roosii Nakaike*	20-week-old male SD rats	Enhance angiogenic-osteogenic coupling by promoting angiogenesis through the PDGF-BB/VEGF/RUNX2/OSX signaling axis	Improve angiogenic capacity and osteogenic capacity of BMSCs	Shen et al. (2022b)
Baohuoside I	*Epimedium brevicornu Maxim.*	Mouse BMSCs cells	Increase ALP activity in OB; Inhibit IL-1b, IL-6, IL-8, and TNF-α secretion	Stimulate differentiation of BMSCs toward OB and inhibit lipid formation; Modulate immune function	Xi et al. (2019)
Luteolin	*Lonicera japonica Thunb.*	1.5-month-old SPF-grade female ovariectomized SD rats	Regulate PI3K-Akt signaling pathway activity	Promote differentiation of BMSCs to OB	Liang et al. (2022)

In this part, the mechanisms through which TCM flavonoids intervene in BMSCs are clarified and the outcomes mainly involve promoting differentiation towards OBs to regulate bone homeostasis. BMSCs proliferate and differentiate into osteoblastic precursor cells and further differentiate into mature OBs. The classical Wnt signaling pathway plays an important role in the survival and differentiation of OB. Therefore, this classical pathway will be the primary entry point in the study of TCM flavonoids for regulating BMSCs differentiation. Based on this pathway, extending to other related signaling pathways (such as NF-κB/β-catenin) or investigating the effects of flavonoids as dietary supplements [such as soy isoflavones (Ge et al., 2023)] on the pathway would be meaningful. Additionally, it has been reported that Wnt signaling pathway, as a key signaling pathway for osteogenic differentiation of BMSCs, is also closely related to the development of hypoxa-induced diseases (Chavali et al., 2020). Bone injuries are usually accompanied by tissue oedema and haematoma, leading to the formation of a local hypoxic environment. Thus, the potential of TCM flavonoids to preserve the osteogenic differentiation capability of BMSCs in hypoxic conditions warrants further research. In addition to this, leveraging existing results and knowledge of relevant signal transduction, forthcoming inquiries could focus on optimizing flavonoid compounds' effectiveness on target cells, introducing other functional proteins or non-coding RNAs into the pathway, facilitating cytokine transmission and controlling cell activation, improving biomarker sensitivity and specificity, and using special medications to mitigate stem cell transplant rejection reactions, which may constitute new avenues of research.

### 3.2 Balancing the bone immune system

Bone homeostasis is associated with the regulation of skeletal system/immune system balance. The concept of osteoimmunology, first introduced in 2000, highlights the close connection between the two systems (Arron and Choi, 2000). Both bone tissue and immune cells originate from the bone marrow, with bone tissue and immune microenvironment influencing each other in physiological and pathological conditions (Lorenzo et al., 2008). The abnormal activation of the immune system can alter the coupling balance of OB and OC, leading to imbalanced bone homeostasis. Concurrently, bone metabolism also regulates the immune system through cytokines, signaling pathways, and estrogen (Terashima and Takayanagi, 2018).

Immune cells produce a variety of cytokines that regulate the production and activity of OB and OC through RANKL-dependent or -independent pathways, influencing bone homeostasis. These cytokines include interleukin-8 (IL-8) (Niu et al., 2019), IL-18 (Mansoori et al., 2016), IL-33 (De Martinis et al., 2020a), IL-34 (Xu et al., 2021), IL-37 (Ye et al., 2019), transforming growth factor-β (TGF-β) (Cao et al., 2022) (bone protective factors), IL-1α (Zheng et al., 2021), IL-1β (Otsuka et al., 2023), IL-6 (Wang et al., 2021), IL-17 (Roberts et al., 2022), IL-31 (De Martinis et al., 2020b), TNF-α (Yao et al., 2021), macrophage-stimulating factor (M-CSF) (Inoue et al., 2023), interferon-γ (IFN-γ) (Biros et al., 2022) (bone resorptive factors), and IL-10, IL-27 (Shukla et al., 2017) (pleiotropic factors). TGF-β is a well-studied cytokine that participates in the production of regulatory cells and exerts immunosuppressive functions to maintain immune homeostasis. In maintaining bone homeostasis, it regulates the replication and differentiation of chondrocytes, OBs and OCs, which play crucial roles in bone formation and mineralization. Furthermore, the TGF-β superfamily has a significant impact on the bone structure (Omosule et al., 2023). OPG/RANKL/RANK is a major signaling pathway mediating bone resorption/bone formation. The equilibrium of RANKL and OPG determines the balance of bone homeostasis. RANKL, expressed by BMSCs and OB, binds to RANK, promoting OC differentiation and enhancing bone resorption activity. On the other hand, OPG, produced by B lymphocytes and OB, competes with RANKL to bind RANK. Therefore, the extent to which OPG interferes with the binding between RANKL and RANK determines the rate of bone resorption. Several lymphocytes are involved throughout the production process and pathway regulation (Song et al., 2020; Frase et al., 2023). Thus, it can be deemed that the signaling pathway serves as a link between various OBs, immune cells, and cytokines, influencing the formation and differentiation of OBs and OCs. Similarly, estrogen also affects the skeletal system/immune system balance. Estrogen receptor (ER) is located on the surface of both OBs and OCs and stimulates OBs directly to regulate differentiation and proliferation, matrix mineralization, and mechanical stress response. In addition to affecting receptors, estrogen also contributes to the development of OP by influencing the body’s inflammation level (Alippe and Mbalaviele, 2019). Chronic inflammatory factors promote bone resorption while inhibiting bone formation. Low estrogen is predisposed toward a low-level chronic inflammatory state, resulting in elevated TNF-α, IL-1, IL-6, IL-17 and decreased expression of IFN-γ, IL-4, and IL-10 (Cheng C. H. et al., 2022; Azam et al., 2023). Previous studies have examined estrogen and inflammation separately with respect to OP; however, accumulating evidence suggests an interrelation between these two mechanisms. Estrogen may also influence OC activity by regulating inflammatory factors, providing a new theoretical basis for the development of OP.

Furthermore, it could be speculated that the essence of bone immune system balance is that different immune cells and cytokines regulate OBs and OCs through classic pathways, such as OPG/RANKL/RANK to promote or inhibit bone resorption and ultimately achieve bone homeostasis. Many TCM flavonoids possess anti-inflammatory and immune-enhancing properties and directly regulate bone immune homeostasis. This process is closely associated with the targets mentioned above. Icariin is the most common among the numerous TCM and active metabolites that promote bone formation (Wang Z. et al., 2018). A previous study designated icariin-treated the bone microvascular endothelial cells (BMECs) as the experimental group and used ultracentrifugation to isolate extracellular vesicles from this group and the blank control group (Zhang et al., 2022). The results revealed significantly increased expression of vascular endothelial growth factor (VEGF) and TGF-β1, and these contents packaged within the vesicles were proposed to promote angiogenesis. The assay also detected differential expression of up to 29 inflammatory factors, primarily involved in OC generation, indicating a dual role of icariin in regulating bone homeostasis through the immune pathway. In addition, epimedium plants contain other flavonoids, including icariin A, B, C, and baohuoside I (BHS I), that exhibit anti-OP activity. In one study, Xi et al. (2019) conducted experiments on BHSI. *In vitro*, the positive control group was treated with genistein, a drug that has been shown to be effective, and the experimental group was treated with different concentrations of BHSI. ALP and TRAP staining revealed that a low concentration (1 μmol/L) of BHSI promoted osteogenic differentiation and inhibited adipogenic differentiation. Meanwhile, *in vivo*, they examined serum inflammatory factor levels in the BHSI-treated de-ovulated rat model and found significant reductions in IL-1β, TNF-α, IL-6 and IL-8. This indicates that BHSI modulates abnormal immune functions. In addition, they critically verified the estrogen-like effects of BHSI due to its structural similarity to icariin. However, it turned out that BHSI did not react with ER-α and ER-β *in vitro*. In terms of inhibiting inflammatory system responses, linarin, a key metabolite of TCM Chrysanthemum indicum L. *[Asteraceae;* chrysanthemi indici flos] and Buddleja officinalis Maxim. *[Scrophulariaceae;* buddlejae flos], was identified by Yang et al. (2022) to reduce the levels of NF-κB, p65, IKKβ, IL-6, and TNF-α. And among the studies on signalling pathways, OPG/RANK/RANKL is undoubtedly the most representative. Hyperoside is present in many botanical drugs and can be extracted from quercetin. Experimental evidence suggests that hyperoside promotes bone formation and reduces bone resorption by inactivating the Traf 6-mediated RANKL/RANK/NF-κB pathway, thereby increasing the OPG/RANKL ratio (Chen et al., 2018). In another study, Jiang et al. (2018) experimented with the epimedium-derived isomeric flavonoids CIT and IT. Validated by molecular docking and animal experiments, they found that the combination of isomeric flavonoids (CIT/IT) with OPG/RANKL targets attenuated the excitation effect of OPG or RANKL on RANK. Moreover, since CIT binds stronger to RANKL than IT, CIT has a more significant ability to inhibit bone resorption. Over all, these findings show the effects of TCM flavonoids on immune cells, cytokines, and signaling pathways. Introducing the variable “estrogen” in studies on flavonoids to regulate the bone immune system would aid in comprehending the effects of TCM on postmenopausal conditions characterized by low estrogen levels and decreased immune function. For instance, molecular docking was used to confirm that chrysin, an extract derived from *Oroxylum indicum (L.) Kurz* in the family Bignoniaceae, interacts with both α and β estrogen receptors with exothermic binding energies of 229.83 kcal/Mol and 252.72 kcal/Mol, respectively, and also fits perfectly into the active site of both α and β estrogen receptors (Ibrahim et al., 2021). The interaction between the drug and the receptor reversed the reduction in estradiol caused by ovariectomy. These metabolites have phytoestrogenic effects that act by binding to the estrogen receptor. Phytoestrogens are a good alternative to hormone replacement therapy. The use of natural phytoestrogens can, to some extent, avoid the adverse effects of long-term administration of exogenous oestrogens, such as breast cancer and endometrial cancer. These drugs are useful in the treatment of OP in postmenopausal women and deserve special attention. For example, among several soybean isoflavones, genistein has the most positive effects on bone cells without any significant adverse effects on the breast and uterus cells due to its high affinity for ER-β receptors compared to ER-α(Arcoraci et al., 2017). Specific studies related to TCM flavonoids balancing the bone immune system are summarized in Table 2.

**TABLE 2:** Studies related to the balancing of skeletal immune system by TCM flavonoids.

Compound	Source	Model	Mechanism	Result	References
Icariin	*Epimedium brevicornu Maxim.*	Male SD rats	Inhibit the RANKL-p38/ERK-NFAT pathway	Inhibit OC differentiation	Cheng et al. (2022b)
		Bone marrow-derived macrophages; rat BMSCs	Promote macrophage polarization toward anti-inflammatory M2 type; Promote osteogenic differentiation of BMSCs and inhibit RANKL-induced OB formation by modulating macrophage cytokines	Modulate host immune response; promote osseointegration	Chai et al. (2022)
		Isolated BMECs in the femoral head of patients with primary and secondary OP	Promote VEGF and TGF-β1 expression in isolated EVs; Differentially express 29 inflammatory factors	Enhance the role of EV in reversing glucocorticoid-induced injury in BMECs	Zhang et al. (2022)
Baohuoside I	*Epimedium brevicornu Maxim.*	Female ovariectomized SD rats	Downregulate IL-1β, TNF-α, IL-6, and IL-8	Inhibit bone resorption	Xi et al. (2019)
Linarin	*Chrysanthemum indicum L.*	2-month-old C57BL/6 male mice	Downregulate MDA; Downregulate NF-kB, p65, IKKβ, IL-6, and TNF-a	Downregulate RANKL/RANK to reduce OC generation	Yang et al. (2022)
Hyperoside	*Hypericum perforatum L.*	68-week-old female ovariectomized Kunming mice	Inhibit TRAF-6-mediated RANKL/RANK/NF-κB pathway and increase OPG/RANKL ratio	Increase bone density and restore bone trabecular microarchitecture	Chen et al. (2018)
		8-week-old female ovariectomized C57BL/6 mice	Downregulate miR-19a-5p-mediated IL-17A levels	Relieve bone resorption	An et al. (2023b)
Chrysin	*Oroxylum indicum (L.) Kurz*	3-month-old female ovariectomized Wistar rats	Interact with ER-α and ER-β	Reduce bone remodeling marker alterations and increase bone mineral content	Ibrahim et al. (2021)
Oroxylin A	*Oroxylum indicum (L.) Kurz*	6-week-old C57BL/6J mice	Reduce ROS levels through Nrf2-mediated antioxidant responses; Attenuate NFATc1 activity	Inhibit RANKL-induced OC activity; Prevent OVX-induced and LPS-induced bone loss	Xian et al. (2021)
Corylin	*Psoralea fructus*	8-week-old C57BL/6 mice	Inhibit P65 nuclear translocation; Attenuate NF-kB and NFATc1 activity	Inhibit OC differentiation	Yu et al. (2021)
Total flavonoids of *Rhizoma drynariae*	*Cullen corylifolium (L.) Medik.*	Murine BMSCs, MC3T3-E1 cells subclone 14, IDG-SW3 cells, RAW 264.7 cells	Modulate Wnt/β-catenin pathway and OPG/RANKL/RANK axis by combined metformin	Improve bone mineral density, bone trabecular microstructure, and mechanical properties	Jiang et al. (2023b)

The emergence of osteoimmunology emphasizes the involvement of the immune microenvironment in the pathological development of OP. This process is regulated by the immune system, which includes bone formation and resorption. TCM flavonoids regulate skeletal system/immune system homeostasis by interfering with cytokines, signalling pathways and estrogen. The OPG/RANK/RANKL pathway, recognized as a key pathway, has been the focus of some studies searching for flavonoids that can specifically regulate it. Flavonoids targeting RANKL or its receptor RANK could open new treatment avenues for OP, particularly in inhibiting excessive bone resorption. An example is the isomeric flavonoid (CIT/IT) mentioned above. Furthermore, it is worth noting that monoclonal antibodies (mAbs) generated by B cells play a considerable role in the treatment of OP. For instance, RANKL mAbs like denosumab inhibit bone resorption, whereas sclerostin mAbs like romosozumab promote bone formation. In terms of the connection between flavonoids and mAbs, the hotspots of research at this stage mainly focus on “adding specific flavonoids to promote cell culture to increase the production of mAbs (Toronjo-Urquiza et al., 2020; Noguchi et al., 2023)” and “producing specific mAbs for flavonoids and establishing immunoassay methods (Zhang et al., 2023).” Although this research is in its early stages, it shows promising potential and merits further investigation.

### 3.3 Inhibiting oxidative stress response

The oxidative/antioxidative balance directly influences bone homeostatic regulation, while estrogen deficiency has been traditionally viewed as the primary cause of OP. Recent mechanistic studies suggested significant pathogenic roles of aging and elevated reactive oxygen species (ROS). Oxidative stress arises from an imbalance between oxidation and antioxidation within the body, wherein ROS production surpasses the buffering capacity of the antioxidant defense system, leading to cellular damage and apoptosis. In the regulation of bone homeostasis, oxidative stress reduces OB activity, further decreasing bone mass and promoting OC production, which causes an imbalance in bone mass. The former may be related to P13K (Ding et al., 2016) and ERK5 (Xia et al., 2017) pathways; the latter might be closely linked to the high expression of NF-κB (Li et al., 2018). In a recent study, *DDIT3* and *FOXO3* were identified as biomarkers of oxidative stress in postmenopausal OP by bioinformatics analysis (Liu et al., 2023). Subsequently, a TF-miRNA-mRNA network associated with these two characterized genes was constructed, offering potential biological targets for future clinical treatment. In addition, oxidative stress can also activate cellular autophagy signaling pathways. Under normal physiological conditions, cells maintain a basal level of autophagy to preserve homeostasis by eliminating damaged organelles. However, under stress conditions, autophagy is upregulated, leading to organelle degradation and induction of apoptosis. In bone tissue, autophagy influences the survival and differentiation of OBs and OCs, regulating oxidative/antioxidative balance and affecting bone homeostasis. In another study, OBs were treated with the autophagy blocker 3-MA and the autophagy activator RAP, respectively (Li et al., 2017). Flow cytometry analysis revealed that autophagy partially mitigated the damage induced by oxidative stress and promoted OB proliferation. However, excessive autophagy activated the apoptotic pathway, leading to OB apoptosis. Apparently, not only oxidative stress has a bidirectional regulatory effect on autophagy, but also autophagy has a bidirectional regulatory effect on bone metabolism.

The mutual antagonism between oxidative/antioxidative responses and the activation of autophagy in senescent cells directly affects the balance of bone homeostasis. Flavonoids, such as soy isoflavones, icariin, silymarin, and other botanical drug extracts, are well-known natural antioxidants with potent anti-inflammatory properties as well as strong antioxidant activity and reducibility. Compared to clinical first-line use, studies have shown that chrysin and quercetin are superior to alendronate as preventive agents on a number of criteria (Oršolić et al., 2014). Several years later they further found that treatment with chrysin or naringenin improved bone quality, reduced bone resorption, and bone mineral deposition, although with a lower efficacy compared with alendronate (Oršolić et al., 2022). However, flavonoids exhibited more pronounced antioxidative, anti-inflammatory and phytoestrogenic activities. These metabolites inhibit the production of ROS radicals by directly blocking free radical chain reactions through the phenolic hydroxyl groups in their structure and promote the decomposition of accumulated free radicals by enhancing the activity of endogenous antioxidant enzymes. Therefore, studies have also investigated the regulation of bone homeostasis by TCM flavonoids through this pathway. Additionally, the studies on dihydroflavone compound silymarin demonstrated its ability to enhance serum total antioxidant capacity (TAC) and SOD2 production while reducing MDA production, thereby decreasing ROS levels and restoring bone homeostasis (Tao et al., 2022). SOD is an antioxidant enzyme that scavenges free oxygen radicals, while MDA is a cytotoxic lipid oxidation product. Naringenin and chrysin, two commonly occurring flavonoids, are also derived from natural TCM plants. The former is widely detected in *Citrus × aurantium f. aurantium* of the Rutaceae family, and the latter is most common in *Oroxylum indicum (L.) Kurz* of the Bignoniaceae family. These two drugs can be extracted through alcohol extraction, chromatography and crystallization. Overall, the regulatory system of bone homeostasis can be considered an interconnected network, wherein individual balances interact and influence each other. In addition to studying the effects of TCM flavonoids on oxidative stress and autophagy alone, recent studies have combined this balance with skeletal system/immune system balance and iron metabolism balance. For instance, a Chinese study assessed the widely used drug icariin in autophagy (Bai et al., 2023). The researchers incubated BMSCs with 100 μM H2O2 to construct a model of senescence state and used 3-MA as a control. They found that icariin at a concentration of 0.1 μM significantly enhanced the expression of autophagic-related genes and activated autophagy in senescent macrophages and rejuvenates osteogenesis of senescent BMSCs. The study also provided insights into the transcriptomic analysis, revealing the significant association of the TNF-α signaling pathway with the level of autophagy, further supporting the concrete data on the mechanism of action of icariin in regulating autophagy and reducing inflammaging.

Additionally, a study reported for the first time the mechanism of quercetin in treating OP caused by iron overload (Xiao et al., 2023). In this experiment, quercetin characteristically reduced apoptosis and ROS production induced by ferric ammonium citrate (FAC). It upregulated Bcl-2 while downregulated the expression of caspase 3 and Bax. These effects were achieved through activating the Nrf2/HO-1 signaling pathway and attenuating FAC-induced oxidative stress damage. In addition to conventional drug intervention targets, recent studies have also focused on combining flavonoid metabolites with other materials to create novel coatings. Based on mussels, Yang et al. (2023) loaded baicalein (BAI) onto bovine serum albumin (BSA) and added tannic acid (TA) to prepare a TA/BAI-BSA composite protein that scavenges ABTS+ and DPPH + free radicals, exhibiting significant antioxidant effects. Tao et al. (2022) modified hydroxyapatite coatings with silybin, a major component of silymarin, to study the effects of interfering with oxidative/antioxidant balance on osteogenic differentiation under high glucose conditions. The findings indicated that silybin restores OB function by activating the SIRT1/SOD2 pathway. The specific studies related to TCM flavonoids for inhibiting oxidative stress response are summarized in Table 3.

**TABLE 3:** Studies related to the inhibition of oxidative stress by TCM flavonoids.

Compound	Source	Model	Mechanism	Result	References
Genistein	*Glycine max (L.) Merr.*	12-week-old female ovariectomized SD rats	Target ER-a to inhibit p16^INK4a^ expression; upregulate SIRT3 and PGC-1a expression	Rescue BMSCs from premature senescence; Elevate ROS levels and mitochondrial dysfunction	Li et al. (2023)
Icariin	*Epimedium brevicornu Maxim.*	12-week-old female ovariectomized BALB/c mice	Upregulate autophagy-related genes *LC3B/A*, *Atg5*, *Atg7* expression and downregulate autophagy marker P62 expression; Activate the TNF pathway	Activate senescent macrophage autophagy and OC differentiation	Bai et al. (2023)
Quercetin	*Styphnolobium japonicum (L.) Schott*	MC3T3-E1 cells	Activate the Nrf2/HO-1 pathway	Counteract iron overload-induced oxidative stress damage	Xiao et al. (2023)
Silymarin	*Silybum marianum (L.) Gaertn.*	12-week-old female ovariectomized SD rats	Promote serum TAC and SOD2 production, reduce MDA production and lower ROS levels	Promote SIRT1 and SOD2 transcription in iron-overloaded MC3T3-E1 to restore bone metabolic homeostasis	Tao et al. (2022)
Baicalein	*Scutellaria baicalensis Georgi*	MC3T3-E1 cells	Remove ABTS+ and DPPH free radicals	Reduce H_2_O_2_ oxidation-induced intracellular ROS to maintain oxidative stress viability	Yang et al. (2023)
Silibinin	*Silybum marianum (L.) Gaertn.*	3-month-old female SD rats; MC3TE-E1 cells	Activate the SIRT1/SOD2 pathway and reduce ROS levels	Improve OB activity and enhance osseointegration	Tao et al. (2022)
Corylifol A	*Cullen corylifolium (L.) Medik.*	9-week-old female ovariectomized SPF mice	Enhance the expression of antioxidant enzyme genes *Cat*, *Hmox1*, and *Nqo1*; Promote the production of antioxidant enzymes CAT and NQO1	Clear ROS and inhibit OC differentiation	Xu et al. (2023)
Galangin	*Alpinia officinarum Hance*	3-month-old male C57BL/6 mice; human BMSCs	Enhance the PKA/CREB signaling	Induce autophagy; Enhance osteogenic differentiation	Zeng et al. (2023b)
Neobavaisoflavone	*Cullen corylifolium (L.) Medik.*	Mouse MC3T3-E1 cells	Activate the CRNDE-mediated Nrf2/HO-1 pathway	Reverse hormonal effects on apoptosis; Affect ROS, MDA, LDH, and Nrf2 levels to protect OB from oxidative stress	Zhu et al. (2021)
Total flavonoids of Rhizoma drynariae	*Drynaria roosii Nakaike*	Aged female ovariectomized SPF rats	Increase SOD and GSH-Px activity and reduce ROS and MDA production	Improve antioxidant defenses; Increase bone density and reduce bone mineral loss	Mu et al. (2021)

The pathological mechanisms of OP are complex, encompassing oxidative stress and autophagy. Therefore, whether flavonoid botanical drugs should be used as antioxidants alone or in combination with other treatments requires further investigation. Besides understanding the mechanisms through which flavonoids influence oxidative/antioxidant balance and autophagy homeostasis, current research is focused on the impact of external factors such as estrogen, glucocorticoids, hyperglycemia, and hypoxia. Additionally, in the field of biomaterials preparation, the application of TCM flavonoids composite implants in bone restoration and the development of novel coatings and scaffolds provide research directions for the future.

### 3.4 Reversing iron overload

Iron overload disrupts iron metabolic homeostasis, leading to death and risk of bone homeostatic imbalance. Iron death is characterized by iron-dependent lipid peroxidation, ROS-induced cell membrane rupture, and mitochondrial miniaturization. A correlation has been established between iron metabolic homeostasis and the oxidative/antioxidant balance. Excessive free Fe^2+^ reacts with lipid peroxides through Fenton reaction, resulting in high levels of ROS accumulation that lead to bone loss (Stockwell et al., 2017; Agidigbi and Kim, 2019). In terms of bone homeostasis, iron overload can inhibit OB activity, interfering with its differentiation and mineralization processes and activating OC activation, causing bone loss and leading to OP. The degree of iron overload is positively correlated with the development of OP (Balogh et al., 2018). In a study on OB, Cen et al. (Luo et al., 2022) concluded that iron death caused by iron overload inhibits OB differentiation via FAC. Mechanistically, this process is achieved by downregulating the expression of Wnt target genes, interfering with the transcription of the Wnt reporter gene *TopFlash* and inhibiting the classical Wnt signaling. A study on OC revealed that iron stimulates OC production in bone marrow-derived macrophages, which is mechanistically dependent on ROS production and activation of the NF-κB signaling pathway (Wang X. et al., 2018). In addition to the effects of iron overload, iron deficiency can also lead to bone loss. Some studies established an iron-deficient environment by chelating iron in the medium using desferrioxamine. These also showed that the effect on OB exhibits a biphasic response. Specifically, cell metabolism is enhanced in mild iron deficiency, while in severe iron deficiency, cell activity is greatly suppressed (Zhao et al., 2012).

Iron death directly affects bone homeostasis, primarily caused by iron overload, which is often treated with iron chelators that facilitate iron excretion, eliminate free iron, reduce iron accumulation in tissues, and alleviate bone loss (Bordbar et al., 2019). The approved iron chelators, deferoxamine, deferiprone, and deferasirox, are effective but have some side effects. Flavonoids have gained increasing attention due to their exceptional iron chelation, antioxidant, and low toxicity properties; hence, several studies have investigated their potential as plant-based iron chelators. As an example, quercetin-treated dendritic cells exhibit gene activation that enhances extracellular iron export and mitigates inflammatory response, thereby reducing intracellular iron levels. Another study proposed that quercetin affects hepcidin expression via the BMP6/SMAD4 signaling pathway, mitigating ethanol-induced iron overload-induced liver injury. In the treatment of orthopedic diseases, naringenin and icariin have been proven to attenuate cartilage damage and promote chondrocyte proliferation and differentiation in the iron overload state (Wang et al., 2016; Pan et al., 2022). Evidently, specific flavonoids from TCM positively regulate iron homeostasis, with research extending to orthopedic conditions, such as arthropathy. Table 4 provides specific information on regulating OP by reversing iron overload. The research in this field is yet in the early stages, with limited publications and depth of exploration compared to other balances. Icariin is one of the most popular medications that has been shown in numerous trials to relieve OP and promote bone regeneration. Yao et al. (2019) used a 100 μM FAC-treated model to simulate iron overload and observed that epimedium glycosides at concentrations of 0.1, 1, and 10 μM significantly enhanced the expression of Runx2, OPN, and β-catenin proteins, identifying 1 μM as the optimal concentration. Meanwhile, icariin safeguarded BMSCs against the collapse in mitochondrial membrane potential (MMP) due to iron overload and mitigated FAC’s inhibitory impact on JC-1 aggregate formation. In addition, icaritin, the glycosidic ligand of the other two metabolites (icariin, baohuoside I) of the *Epimedium brevicornu Maxim.* plant, has adequate musculoskeletal permeability and iron complexing properties. It can also utilize unstable plasma iron to construct artificial iron pools that would re verse the iron overload and promote osteogenesis (Jiang J. et al., 2023). Icaritin was hypothesized to act as a potential natural phytochelator in this experiment. The regulatory processes of TCM flavonoids are directly related to the balance of oxidative/antioxidative systems. Iron overload leads to an excess of lipid ROS free radicals, leading to the accumulation of lipid hydroperoxides and disruption of cell membrane structure, which ultimately triggers iron death.

**TABLE 4:** Studies related to reversal of iron overload by TCM flavonoids.

Compound	Source	Model	Mechanism	Result	References
Icariin	*Epimedium brevicornu Maxim.*	MC3TE-E1 cells; 5-week-old C57BL/6 mice	Promote OB survival; Reverse iron overload-induced decrease in Runx2, alkaline phosphatase and bone-bridging protein expression	Reverse excess iron ion-induced bone loss	Jing et al. (2019)
		Male SD rats	Regulate mitochondrial fusion and division; Activate the PI3K/AKT/mTOR pathway and inhibit the ERK1/2 and JNK pathways	Reduce damage to BMSCs from iron overload	Yao et al. (2019)
Icaritin	*Epimedium brevicornu Maxim.*	Female Kunming mice, female SD rats; zebrafish embryos	Complex-free iron to construct AIP	Reduce iron overload and low-bone density promotes osteogenesis	Jiang et al. (2023a)
Quercetin	*Styphnolobium japonicum (L.) Schott*	MC3TE-E1 cells	Increase ALP activity to promote bone mineralization nodule formation; Upregulate Runx2, Osterix, and Bcl-2 expression; Downregulate caspase 3 and Bax expression; Activate the Nrf2/HO-1 pathway	Attenuates FAC-induced oxidative stress damage, iron deposition, and bone loss	Xiao et al. (2023)
Silymarin	*Silybum marianum (L.) Gaertn.*	12-week-old female ovariectomized SD rats	Promote OB proliferation and differentiation and increase OB ability to secrete ALP and mineralization; Enhance the transcription and expression levels of target genes, such as *Runx-2*, *SOD2*, and *SIRT1*	Improve bone metabolism and oxidative stress status	Tao et al. (2022)

In conclusion, iron chelators constitute a potential therapeutic strategy in animal models. Iron overload and the phenomenon of iron death causing OP have also become a hot topic of research. Nonetheless, in-depth studies would utilize the unique advantages of TCM flavonoids in addition to their chelating, oxidizing, and low side effects. For instance, future research would focus on investigating the role of various hormones (EPO and iron modulators) and the impact of external factors, such as inflammation, acidosis, and anemia. Notably, in recent years, our understanding of the mechanism of iron death has improved, and another theory known as “copper death” has gained significant attention. Copper death is distinct from iron death and oxidative stress in terms of the underlying mechanism that triggers OP. The phenomenon is closely related to immune factors and directly mediates the skeletal system/immune system balance (Xu et al., 2018; Liu et al., 2022). Similarly, TCM plants have significant potential for the study of copper death on OP. For instance, copper oxide quantum dots were successfully synthesized on chitosan with the aid of *Angelica sinensis*, resulting in enhanced OB activity and alleviating OP(Hu et al., 2019).

## 4 Conclusion and future perspectives

TCM has a long history in the treatment of OP. Presently, bone-active metabolites contained in TCM have become the focus of OP research, with pharmacologists exploring their mechanisms of action. The treatment of OP is complex, with equal importance placed on inhibiting bone destruction and enhancing bone formation. In clinical practice, combining multiple drugs, including sequential therapy, is commonplace. With its unique multi-target and multi-pathway advantages, TCM can effectively treat complex diseases with lower dosages. At the same time, TCM can also be taken in the form of dietary therapy, which can overcome the drawbacks and adverse effects of long-term administration of conventional drugs. Human diets not only contain six major nutrients but also non-nutrient components such as flavonoids. Flavonoids play a therapeutic and regulatory role in bone health by affecting the body’s absorption of minerals such as calcium and magnesium, as well as the synthesis of collagen (a major component of the bone matrix). Moreover, from the perspective of TCM, it is common to use of several herbal plants’ mixtures or drug pair as a therapeutic option. So using flavonoids as a dietary supplement has great potential and it will help to avoid the damage caused by first line drugs’ side effects. However, we must also recognize that current research is still largely focused on cellular and animal experiments. Screening for efficient TCM flavonoids for new drug development and their early clinical validation and use is greatly beneficial for the advancement of this field.

Herein, this paper categorises and summarises four different types of balance affecting bone homeostasis, each of which is independent but interrelated. The closest connections were skeletal system/immune system balance, oxidative/antioxidant system balance, and iron metabolism balance, whereas the BMSCs osteogenic/adipogenic differentiation appeared more as a consequence. The therapeutic mechanism of TCM flavonoids for OP also unfolds based on balance. It is through “intervening in the differentiation of BMSCs,” “balancing the bone immune system,” “inhibiting oxidative stress response,” and “reversing iron overload” that drugs characteristically target OBs or OCs, and ultimately re-establish a new bone homeostasis. Exploring pathogenesis from the perspective of bone metabolic homeostasis offers fresh insights into the mechanisms of drug action and the development of new therapeutic targets. Similarly, many balancing systems, such as copper metabolism balance and intestinal flora balance, have homeostatic properties, which have not yet been studied and may be a potential hot spot in the future. In copper metabolism, copper and iron are trace elements in the human body, which can be investigated by analogy based on the existing results of iron metabolism balance, while the intestinal flora balance can be linked to the gut-bone and other balance axes. These phenomena would aid in studying the effects of intestinal flora, microorganisms, and endocrinology on bone homeostasis. Additionally, in terms of signalling pathways, it is not difficult to find that Wnt/β-catenin and OPG/RANKL/RANK are deservedly the star pathways in the study of flavonoids in treating OP, and many recent studies are based on these two. Further research could identify targeted agonists or inhibitors for these pathways. Besides this, the research process on other pathways must be accelerated.

Despite the potential of TCM flavonoids in regulating bone homeostasis, the experimental research faces limitations. 1) The chemical composition of flavonoids extracted from different TCM sources varies considerably, which can affect their biological activity and efficacy in treating OP. Future research could focus on characterizing flavonoid profiles of TCM more precisely to establish standardized extracts for clinical evaluation. 2) Many flavonoids have low bioavailability due to poor absorption, rapid metabolism, and elimination. It is necessary to enhance the bioavailability of flavonoids through means such as biotransformation and novel drug delivery systems to exert their biological activity. 3) Indicators such as therapeutically effective dose, dietary recommendations and maximum tolerable intake are uncertain, and side effect manifestations require long-term follow-up studies. 4) The current studies on flavonoids remain at the level of animal experiments, with only a few clinical experiments, due to the bidirectional regulatory effect and multi-targeting properties of TCM. 5) Apart from studies on single drugs, studies on multi-targets and multi-pathways for the combined use of multiple TCM are still at a low level. There is a need to improve the design of trials and expand the samples of clinical studies. 6) Besides the in-depth research on well-known phytoestrogenic ingredients such as soy isoflavones and corylin, controversies persist regarding phytoestrogen concentrations and the regulation of estrogen receptor genes in OB and BMSCs. Subsequently, estrogen-like activity screening and comparisons of TCM metabolites are needed. 7) Progress in translating research into innovative applications is limited. Future efforts should explore using TCM flavonoids to develop new cellular scaffolds and coatings. These findings expand our understanding of various diseases, such as OP, leading to fractures and bone defects.

In conclusion, TCM flavonoids have promising prospects for development and application with advancing technology. The research in this field will provide new insights for finding effective and safe natural medicines. At the same time we have identified limitations and offered some practical solutions. It is necessary to utilize the advantages of TCM in treating OP, and explore the integration of Chinese and Western medicine. At the same time, focusing on the innovation the dietary therapies and development of new drugs from TCM flavonoids is of great significance for the prevention and treatment of OP.

## Figures and Tables

**Table:** 

**AIP**	artificial iron pools
**ALP**	alkaline phosphatase
**BAI**	baicalein
**BHSI**	baohuoside I
**BMECs**	Bone microvascular endothelial cells
**BMSCs**	Bone marrow mesenchymal stem cells
**BSA**	bovine serum albumin
**ER**	estrogen receptor
**EVs**	extracellular vesicles
**FAC**	ferric ammonium citrate
**IFN-γ**	interferon-γ
**IL**	interleukin
**LDH**	lactate dehydrogenase
**LPS**	lipopolysaccharide
**M-CSF**	macrophage-stimulating factor
**MDA**	malondialdehyde
**OB**	osteoblast
**OC**	osteoclast
**OCN**	osteocalcin
**OP**	osteoporosis
**OPG**	osteoclastogenesis inhibitory factor
**OVX**	ovariectomy
**PKA**	protein kinase A
**ROS**	reactive oxygen species
**SOD**	superoxide dismutase
**TA**	tannic acid
**TAC**	total antioxidant capacity
**TCM**	traditional Chinese medicine
**TGF-β**	transforming growth factor-β
**TNF-α**	tumour necrosis factor-α
**TRAP**	tartrate resistant acid phosphatase
**VEGF**	vascular endothelial growth factor
